# Anomalous mechanical behavior of nanocrystalline binary alloys under extreme conditions

**DOI:** 10.1038/s41467-018-05027-5

**Published:** 2018-07-12

**Authors:** S. A. Turnage, M. Rajagopalan, K. A. Darling, P. Garg, C. Kale, B. G. Bazehhour, I. Adlakha, B. C. Hornbuckle, C. L. Williams, P. Peralta, K. N. Solanki

**Affiliations:** 1 School for Engineering of Matter, Transport, and Energy, Arizona State University, Tempe, AZ 85287 USA; 2 US Army Research Laboratory, Aberdeen Proving Ground, Adelphi, MD 21005 USA; 3 Department of Applied Mechanics, Indian Institute of Technology Madras, Chennai, 600036 India

## Abstract

Fundamentally, material flow stress increases exponentially at deformation rates exceeding, typically, ~10^3^ s^−1^, resulting in brittle failure. The origin of such behavior derives from the dislocation motion causing non-Arrhenius deformation at higher strain rates due to drag forces from phonon interactions. Here, we discover that this assumption is prevented from manifesting when microstructural length is stabilized at an extremely fine size (nanoscale regime). This divergent strain-rate-insensitive behavior is attributed to a unique microstructure that alters the average dislocation velocity, and distance traveled, preventing/delaying dislocation interaction with phonons until higher strain rates than observed in known systems; thus enabling constant flow-stress response even at extreme conditions. Previously, these extreme loading conditions were unattainable in nanocrystalline materials due to thermal and mechanical instability of their microstructures; thus, these anomalies have never been observed in any other material. Finally, the unique stability leads to high-temperature strength maintained up to 80% of the melting point (~1356 K).

## Abstract (web summary)

Metals deformed at very high rates experience a rapid increase in flow stress due to dislocation drag. Here, the authors stabilise a nanocrystalline microstructure to suppress dislocation velocity and limit drag effects, conserving low strain-rate deformation mechanisms up to higher strain rates and temperatures.

## Introduction

A material’s ability to withstand high-deformation rates without failure has a profound effect on many applications. Indeed, in modern industrial society, e.g., in the case of alternating energy supply (fusion reactors), automobile crashes, projectile impact, and deep-space exploration (space debris and meteorite impact), structural metals often experience sudden deformation rates of 10^3^–10^8^ s^−1^ or higher, which in some instances, can lead to catastrophic failures due to loss in ductility not seen at lower strain rates^1–5^. This distinctly different high rate behavior is in part due to dislocation interactions with phonons that arise from lattice vibration within the crystal (commonly known as phonon drag)^3,6–10^, which limits the materials’ ability to deform plastically prior to failure. Thus, engineers have been in a quest to improve the fracture toughness of coarse-grained metals and alloys to prevent such unintended outcomes. In other words, the aim of current research is to determine whether a material can be designed that will deform with a constant flow stress even when subjected to extreme conditions of pressure and high rates of loading^11^. This will require the material plastic response to remain the same under various loading rates. For such behavior to exist, the long-established mechanism of phonon drag leading to flow-stress increase (which is proportional to the loss in ductility) must be prevented from manifesting. The realization of such a material provides a pathway for synthesizing a new-level of tough and high-energy absorbing materials. However, designing a coarse-grained metal or alloy to defy such physics is convoluted by the intensity of the deformation-rate (10^−8^–10^8^ s^−1^).

On the other hand, where the development of coarse-grained metals has fallen short in engineering-enhanced properties, nanocrystalline (NC) metals with an average grain size <100 nm, hold new promise, such as an order of magnitude increase in room temperature strength and superplastic behavior at low temperatures^12^. However, until now, the same types of deviating mechanical responses that have been found to exist in the low strain-rate regime have not been shown to manifest under extreme conditions. In other words, the limited room temperature high-strain-rate studies in NC materials have observed the same dramatic increase in flow stress >10^2^–10^3^ strain per second as in coarse-grained materials^8,10^ due to grain growth.

In the following, we present evidence that, for a material with the average grain size and microstructural length scale decreased and stabilized below 100 nm, the long-established mechanisms associated with phonon drag leading to flow-stress increase are prevented from manifesting^13^. This behavior holds true for temperatures as high as 473 K. The stable mechanical properties are shown to result from a nanostructure full of Ta nanoclusters, which pin both grain boundaries and dislocations. The Ta nanoclusters are shown to undergo no significant change in size or spacing as temperature is increased leading to the notable stability of the mechanical properties. Atomistic simulations show that, while the introduction of Ta in Cu increases the density of phonons within the material, the spacing between the nanoclusters prevents dislocations from achieving the steady-state velocity. This indicates that dislocation interactions with phonons do not contribute noticeably to the strengthening of the material at strain rates up to 10^4^ s^−1^ at 300 K. Previously, extreme temperature and strain-rate regimes were unattainable in NC materials without a loss of the NC structure due to thermal and mechanical instability; thus, neither extraordinary high-temperature strength nor delayed flow-stress increase has ever before been observed.

## Results

### High-strain-rate mechanical responses at 298 K

To prove the existence of such behavior (constant flow stress as a function of strain rate) and to provide a pathway for developing a strain-rate-insensitive material, we prepared a bulk NC-Cu-10 at.% Ta, processed through high-energy cryogenic mechanical alloying followed by consolidation to bulk using equal-channel angular extrusion at 973 K. This process resulted in a microstructure consisting of an average Cu grain size of 50 ± 18 nm, and Ta-based nanoclusters having an average diameter of 3.2 ± 0.9 nm along with an average inter-cluster spacing of 5.23 ± 1.74 nm. These results are comparable to an earlier 3D atom probe study^14^. More details pertaining to material processing conditions are given in the methods section. This alloy was studied previously using limited mechanical testing methods, such as high-temperature creep^15^, where we used this NC alloy to assess the response of a NC material under high temperatures.

**Fig. 1:**
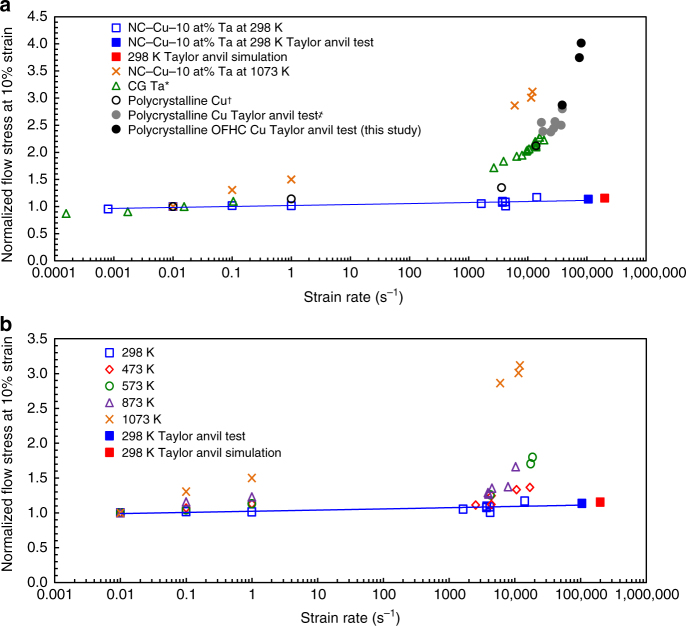
Normalized flow stress as a function of strain rate and temperature. **a** NC-Cu-10 at.%Ta under ambient conditions compared to high-purity Cu and Ta where *, †, and ҂ refer to the results of coarse-grained Ta from ref. ^3^, coarse-grained pure Cu from^9^, and coarse-grained oxygen-free high-conductivity (OFHC) Cu tested by Taylor anvil experiment from ref. ^16^, respectively. **b** NC-Cu-10 at.% Ta at varying testing temperatures. The flow stress at a 10% plastic strain level was taken for each strain rate and normalized by the flow stress at 10^−2^ s^−1^. Data above 10^4^ s^−1^ (solid symbols) was approximated using Taylor anvil experiments. The power law fit for the Cu-10 at.% Ta data reveals that the material is subjected primarily to thermal activation processes up to a strain rate of at least 10^4^ s^−1^

Here, to analyze the effects of temperature, strain rate and microstructure length scale on the macroscopic manifestation of phonon-drag, conventional uniaxial compression tests were performed over a wide range of strain rates (10^−4^–10^5^ s^−1^) and temperatures (223–1273 K) (Methods). Figure 1 shows the flow-stress results at a constant plastic strain of 10%, which are normalized with the 10% flow stress obtained from the same temperature at a strain rate of 10^−2^ s^−1^ then plotted as a function of strain rate. The normalized flow-stress data for polycrystalline Cu^9^, polycrystalline Ta^3^, and coarse-grained oxygen-free high-conductivity Cu obtained by Taylor anvil experiments and those reported in ref. ^16^ are also presented in Fig. 1a with more details in Methods and Supplementary Note 6. A clear upturn in the flow stress, albeit initiating at differing strain rates, can be noted for polycrystalline Cu and Ta. The observed dramatic rise in flow stress (or loss in ductility) is fundamental in origin, and therefore, common to many structural metals such as Cu, Ni, and Ta and believed to be the direct result of the rise in the mobile dislocation drag force brought about by interactions with phonons in the crystal lattice and in some heavily deformed coarse-grained samples due to increase in the dislocation density^3,6–10^. The fundamental theory of phonon drag on mobile dislocation motion has been a seminal contribution in describing the mechanical behavior as a function of strain rates despite microstructural differences for the past 50 years^17,18^. However, this prevailing theory may not be applicable to stable NC alloys as the flow-stress results indicate that for the truly stable NC material reported here, the general behavior of flow-stress increase is absent even up to rates as high as 10^5^ s^−1^ where the flow-stress upturn due to phonon drag can be seen to play a more dominant role in the other material systems reported in literature^8^. For instance, the room temperature flow stress at ~4.0 × 10^3^ s^−1^ for polycrystalline Cu^9^ and Ta^3^ is increased by factors of 6 and 10, respectively, over the NC-Cu-10 at.% Ta which indicates that the NC alloy is minimally influenced by phonon-drag forces. To further support observations of this behavior, we use direct Taylor anvil experiments and simulations to extract approximate material behavior at strain rates closer to 10^5^ s^−1^, see Fig. 1a. Note that experimentally quantifying flow stresses beyond the 10^5^ s^−1^ strain rate is incredibly difficult considering the state of stress or shock-induced effects. Nevertheless, a Taylor anvil experiment coupled with finite element analysis has been employed to qualitatively infer the strength of the material reported here for coarse-grained pure Cu and NC-Cu-10 at.% Ta (Methods). The Taylor anvil experiment at 10^5^ s^−1^ shows a negligible variation in the stress for NC-Cu-10 at.% Ta as compared to the well-agreeing data of coarse-grained Cu from literature and experimentally tested here, asserting the absences of a flow-stress upturn behavior or at the very least, a delayed onset of flow-stress upturn.

### High-strain-rate thermomechanical responses

Given the observed flow-stress behavior as a function of strain rates at room temperature, the response of the strain-rate-dependent behavior as a function of increasing temperature is next to be probed. In general, we know that the classical quantum mechanical description predicts the nature of phonons to obey Bose–Einstein statistics^19^; hence, the mean occupancy number of phonons increases significantly with temperature. Therefore, the temperature dependence of flow stress for NC-Cu-10 at.% Ta was probed for different strain rates as shown in Fig. 1b. The temperature effects reveal that the upturn in the flow stress is delayed even at elevated temperatures, i.e., up to 473 K, where phonons are more prominent as compared to the room temperature, and yet, a negligible dependence persists up to strain rates of ~1.8 × 10^4^ s^−1^. Further, despite increasing the mean occupation of phonons by 3 orders of magnitude^19^, the influence of phonon drag in NC-Cu-10 at.% Ta at 873 K is similar to that exhibited by coarse-grained pure Cu at 298 K. Also, it must be noted that phonon drag is highly rate-sensitive, in that the drag effect increases significantly when the material strain rate is increased by a small fraction beyond a critical rate (generally ~10^3^ s^−1^). For example, as a direct result of a marginal increase in strain rate at 573 K, NC-Cu-10 at.% Ta has attained a similar level of normalized flow stress to when tested at 873 K, despite a 300 K difference in temperature. This observation highlights the significance of the minimal increase in normalized flow stress resulting from the Taylor anvil experiments, e.g., deformation at very high strain rates (10^5^ s^−1^). Eventually, the effects of temperature begin to dominate at 1073 K, and NC-Cu-10 at.% Ta demonstrates a clear upturn, which is indicative of the true non-Arrhenius-dependent regime of deformation.

### Archetype microstructure of a NC alloy

**Fig. 2:**
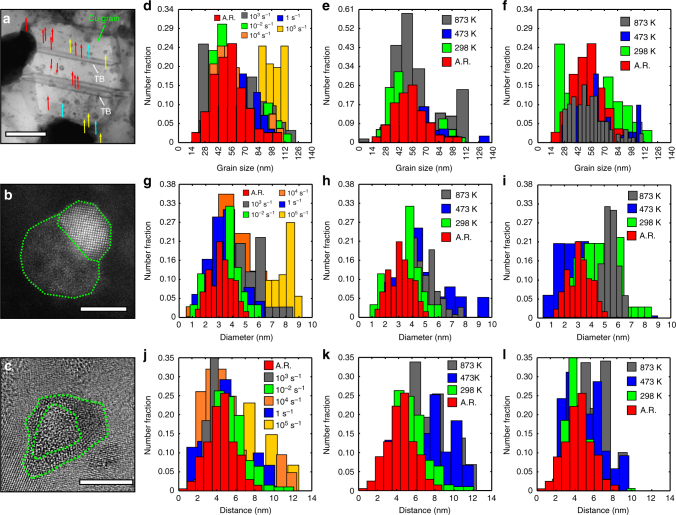
Microstructural characterization of as-received and post-deformed NC-Cu-10 at.% Ta tested at various temperatures and strain rates. **a** Bright-field STEM image indicating the features of as-received NC-Cu-10 at.% Ta such as twins, and the structure and distribution of nanoclusters. The red arrows are for smaller nanocluster sizes followed by green and blue for larger sizes. **b** HR-STEM HAADF image of a co-joined nanocluster with crystalline and distorted components of the nanocluster. The interface between the components and matrix is highlighted in green-dashed lines. **c** HR-TEM image of another type of nanocluster with a core-shell morphology where the shell is crystalline, and the core consists of defects such as vacancies. **d** As-received and post-deformed Cu grain size plotted as a function of strain rate at 298 K. Cu grain size plotted as a function of temperature at a strain rate of **e** 0.01 s^−1^ and **f** 4 × 10^3^ s^−1^. **g** As-received and post-deformed Ta nano-dispersion size plotted as a function of strain rate at 298 K. Ta nano-dispersion size plotted as a function of temperature at a strain rate of **h** 0.01 s^−1^ and **i** 4 × 10^3^ s^−1^. **j** As-received and post-deformed Ta inter-cluster spacing plotted as a function of strain rate at 298 K. Ta inter-cluster spacing plotted as a function of temperature at a strain rate of **k** 0.01 s^−1^and **l** 4 × 10^3^ s^−1^. The white bar in **a** indicates a length scale of 50 nm and that in **b**, **c** indicates a length scale of 5 nm

Following the divergence from classical behavior observed, we turn to further post-deformed microstructural characterizations. Figure 2a–c illustrate the archetype microstructure of the present material, where the nanoclusters (defected core type) are distributed homogeneously along the grain interiors and at grain boundaries (see Supplementary Note 7 and Supplementary Figure 5). As discussed earlier, the average diameter of the nanoclusters was found to be 3.2 ± 0.9 nm with an average effective inter-cluster spacing of 5.23 ± 1.74 nm and a number density of 6.5 × 10^23^/m^3^. These numbers remain nearly constant with increase in strain rate and temperature, highlighting the stability and resistance to coarsening (Fig. 2g–k). Such small changes in the microstructure are very surprising given that the average grain size is <100 nm at the deformation temperature of 873 K. Thus, the stability of the Cu grains (Fig. 2d–f) remains intact owing to the stability of the high density of coherent nanoclusters (Fig. 2a).

**Fig. 3:**
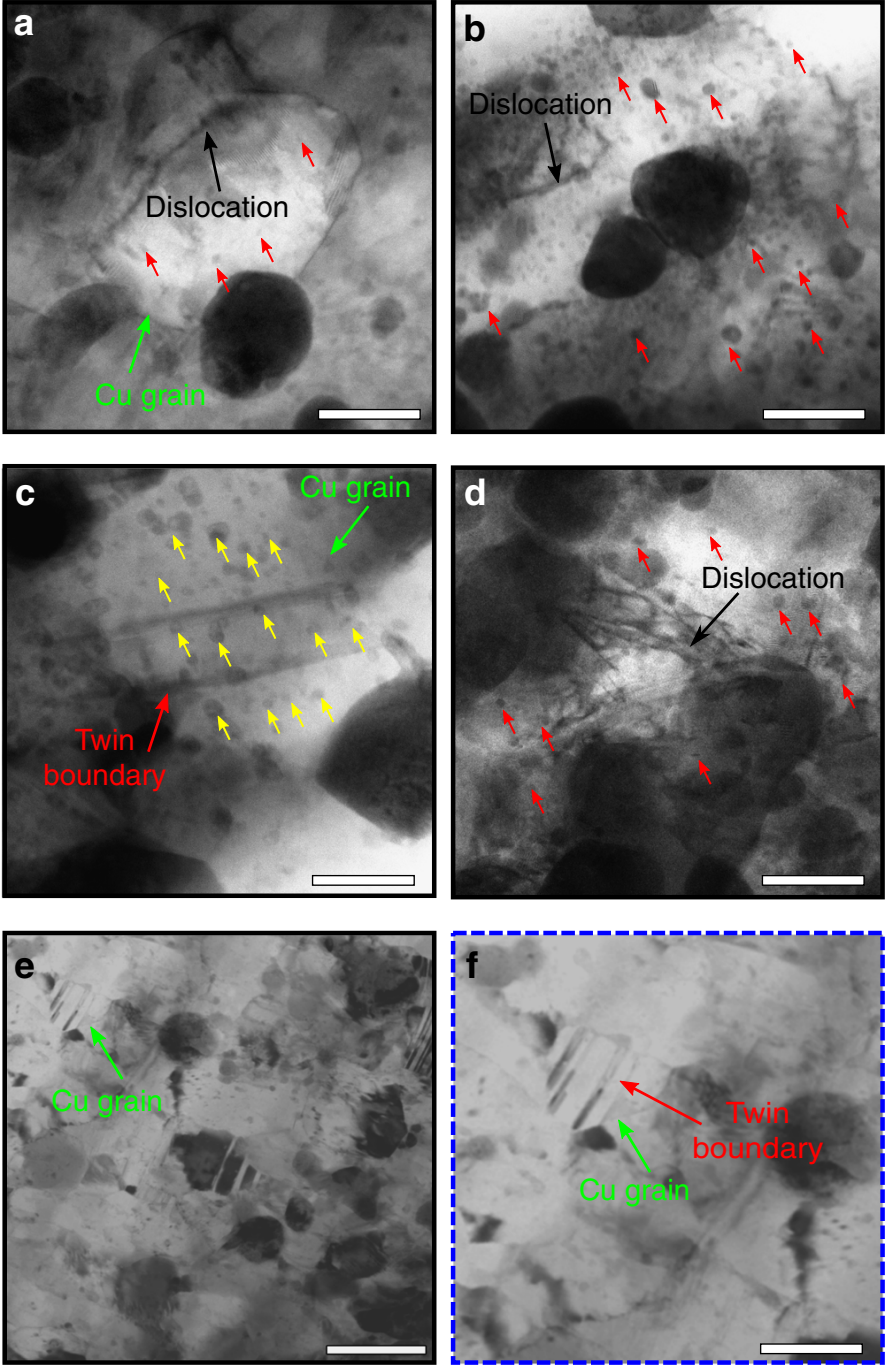
Microstructural characterization of post-deformed NC-Cu-10 at.% Ta tested at multiple strain rates and temperatures. Post-deformed BF-STEM micrograph of NC-Cu-10 at.% Ta at **a** 298 K and 10^−2^ s^−1^, **b** at 873 K and 10^−2^ s^−1^, **c** at 298 K and 4 × 10^3^ s^−1^, **d** at 873 K and 4 × 10^3^ s^−1^, **e** Taylor anvil tested sample, and **f** magnified view of the micrograph in **e** showing twins inside a Cu grain. At lower temperatures (298 K), the high stress contributes toward the nucleation of partial dislocations. At higher temperatures (873 K), emission of dislocations is favored even at 4 × 10^3^ s^−1^ strain rate. The white bars in **a**–**d** and **f** indicate a length scale of 50 nm and that in **e** indicates a length scale of 100 nm

Examination of microstructures after exposure to varying extreme environments reveals negligible changes in the grain size, Ta nanocluster size, and Ta nanocluster dispersion, which raises the question: what is the role of microstructural length scale such as average grain size and nanocluster spacing on the dislocation nucleation and velocity, phonon dispersion/scattering, and overall manifestation of the phonon-drag effects? First, we assert the dislocation interaction with nanoclusters through post-deformed microstructural analyses, which are shown in Fig. 3 and Supplementary note 8. The high-resolution transmission electron microscopy (HRTEM) images of post-deformed samples showing pinning of partial and full dislocations by stable nanoclusters and grain boundaries at various strain rates and temperatures are shown in Fig. 3. At low strain rates (Fig. 3a, b), the deformation is primarily a result of dislocations overcoming the activation energy imposed by grain boundaries and nanoclusters. However, at room temperature and strain rates above 10^3^ s^−1^, the deformation-mechanism shifts from being dislocation dominated to being dominated by twin nucleation. Figure 3 shows the appearance of narrow twins within the microstructure, the spacing of which is reduced when the strain rate increases from 10^3^ to 10^5^ s^−1^ to ~5 nm. By comparison, deformation twins formed in pure NC Cu are reported to be much wider implying that they have undergone growth during loading^20^. However, as seen in Fig. 3c, twin growth (dislocation shear) is impeded by nanoclusters. Furthermore, the average measured twin area fraction through TEM images was found to only be about 4.0% and 5.3% for 10^3^ and 10^5^ s^−1^, respectively, a relatively small change in percentage of the microstructure despite the intense deformation. Additionally, nanoclusters, with an average pinning distance of ~5 nm (Fig. 2 and Methods), reduce the average dislocation velocity of both partial and full dislocations and, as a result, contribute to effectively negating the influence of the phonon-drag force. As temperature increases >873 K, fewer twins and more dislocation clusters are observed (Fig. 3c) implying that the higher temperatures increase thermal activation of dislocations and reduce activation barriers for thermal bypass of nanoclusters. Dislocation bypass is further enhanced by a reduction in the cluster-matrix-coherency strain^21^. Collectively, this loss of pinning efficiency leads to increasing the average dislocation velocity in a lattice environment whereby the drag coefficient is already increased as a result of elevated temperatures. Taken together, these mechanisms correlate to the increased flow stress at high temperatures as the accelerating dislocations begin interacting with an increased number of lattice vibrations resulting in phonon drag.

### Atomistic simulations of deformation behavior

**Fig. 4:**
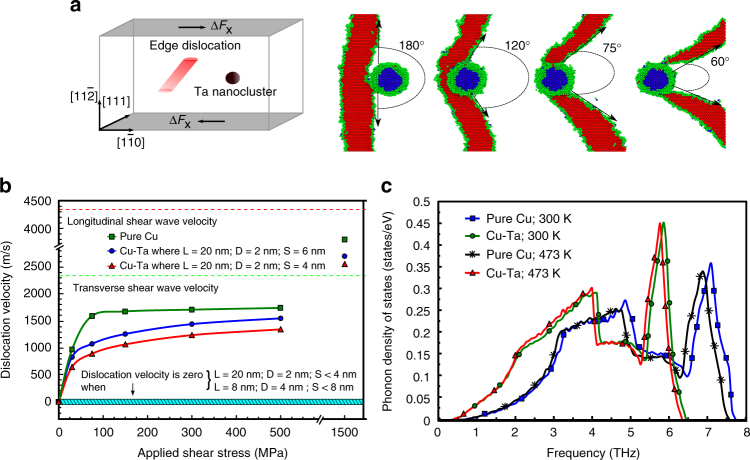
Atomistic simulation of dislocation interaction with a Ta nanocluster. **a** A schematic of the simulation of the interaction between an edge dislocation and a Ta nanocluster in a single Cu nanocrystal where *X*, *Y*, and *Z* correspond to the $\left[ {1\bar 10} \right]$, [111], and $\left[ {11\bar 2} \right]$ directions, respectively, and atomistic simulation results showing an edge dislocation interacting with a Ta nanocluster in a Cu nanocrystal. The red atoms represent the stacking fault region of the dislocation in Cu, the blue atoms represent the Ta nanocluster, and the green atoms represent the boundaries of the crystalline Cu atoms. **b** Dislocation velocity as a function of applied shear stress for an edge dislocation in nanocrystalline Cu both with and without an embedded Ta nanocluster for 100 K. Here, *L* is the dislocation line length, *D* is the diameter of the Ta nanocluster, and *S* is the distance between the dislocation and Ta nanocluster. Note that for 300 K, the dislocation is instantaneously pinned by the nanocluster at values of *S* up to 6 nm without any externally applied stresses. **c** Comparison of phonon density of states for pure Cu and Cu embedded with a Ta nanocluster at both 300 and 473 K

The observed unique strength and strain-rate-independent behavior raises three important questions: what is the role of Ta nanoclusters on strengthening, can a dislocation move fast enough that drag effects become dominant in a NC-Cu–Ta alloy, and what role does phonon-dislocation interaction play in strengthening the NC-Cu–Ta alloy? To address this, atomistic simulations were conducted employing the molecular-dynamics (MD) method (Methods). First, in order to determine the specific contributions of nanoclusters on the strengthening of the NC-Cu-10 at.% Ta, contributions from grain-size effects (Hall–Petch), rule of mixtures, and frictional stresses are removed from the measured flow stress of the material (Methods). The experimentally measured 10% flow stress (σ_y,E_) for  1 s^−1^ strain rate and 300 K is about 1307 MPa and the theoretical estimates for the Hall–Petch (σ_gs_, with average grain size of 57 nm) and rule of mixtures (*σ*_ROM_) contributions were made using Eqs. 1 and 2, respectively.

1 $$\sigma _{{\mathrm{gs}}} = kd^{ - 1/2},$$

2 $$\sigma _{{\mathrm{ROM}}} = V_{{\mathrm{ppt}}}\left( {H_{{\mathrm{ppt}}} - \left( {\frac{{H_{{\mathrm{HP}}}}}{{1000}}} \right)} \right),$$

where *d* is the average grain size, *k* is a material constant for NC-Cu-10 at.% Ta with a value of 0.11 MPa-m^[1/2] 22^, *V*_ppt_ is the volume fraction of Ta nanoclusters, and *H*_ppt_ and *H*_HP_ are the hardness of the Ta nanoclusters (4.1 GPa) and the hardness from the Hall–Petch effect, respectively. The values of these contributions to the flow stress are listed in Table 1. Since the frictional stress in pure Cu is a constant and is about 20 MPa^22^, this leaves the estimated dislocation-nanocluster strength contribution of 786 MPa, which is almost 1.7 times higher than the Hall–Petch effect. For a comparison of Hall–Petch strengthening measurements for varying Ta concentrations in Cu, see Supplementary Note 9. However, it should be noted that these mechanisms may interact with each other, giving rise to a much higher strengthening efficiency. Nevertheless, from atomistic simulations of the interaction between a dislocation with an 8 nm line length and a 4 nm diameter Ta nanocluster (Fig. 4a), the dislocation-nanocluster strengthening (*σ*_dn_) at room temperature was computed to be 867 MPa. The similarity of dislocation-nanocluster contributions between experimental and simulation results indicate that Ta nanoclusters play an important role in strengthening the NC-Cu-10 at.% Ta.

**Table 1:** The various strengthening mechanisms in NC-Cu-10 at% Ta operating under uniaxial compression loading

	*T* = 298 K	*T* = 473 K
*σ*_y,E_ (MPa) experiment	1307	1042
*d* (nm) experiment	57	61
*σ*_gs_ (MPa) *H*–*P* prediction	461	431^a^
*σ*_0_ (MPa) frictional stress	20	20
*σ*_ROM_ (MPa) rule of mixtures	40	40
*σ*_dn_ (MPa) dislocation-nanocluster strengthening: experiment	786	551
*σ*_dn_ (MPa) dislocation-nanocluster strengthening: simulations	867	695

The experimental flow stress values were taken at 10% strain for curves obtained from uniaxial tests at a strain rate of 1 s^−1^

^a^The modified *H*–*P* relationship that includes temperature effects^40^

Next, atomistic simulations were also used to determine the effects of Ta nanoclusters on the average velocity of a dislocation in the Cu matrix. In a copper nanocrystal with no Ta particles (see green line of Fig. 4b), the dislocation velocity increases linearly with applied shear stress up to 100 MPa at 100 K; however, the velocity dependence deviates from the linear trend at stresses above 100 MPa and the velocity converges to an asymptotic value of 1620 m/s, which is below the shear wave velocity (2325 m/s) in copper. At a higher applied shear stress of 1500 MPa, the dislocation velocity transitions from subsonic to transonic with a velocity of 3800 m/s. These results were found to be consistent with the reported theoretical values in literature and confirm the validity of our MD calculations^23,24^. In the presence of a Ta nanocluster, the steady-state dislocation velocity becomes dependent on the dislocation line length (*L*), the size of the Ta nanocluster (*D*), and the temperature all of which influence the distance for the dislocation to travel to the next barrier (*S*). In the atomistic study performed here at 100 K, *L* and *D* were held constant as *S* varied from 4 to 6 nm. Results, shown in Fig. 4b, indicate that at a temperature of 100 K, with a line length of 20 nm and a nanocluster diameter of 2 nm, a value of *S* below 4 nm results in the dislocation becoming absorbed instantaneously with no externally applied flow stress. Further, when the temperature is increased to room temperature (300 K), the dislocation is instantaneously pinned by the nanocluster at values of S up to 6 nm without any externally applied stresses. According to the statistics from Fig. 2j–l, the average Ta inter-cluster spacing is ~4 nm in NC-Cu-10 at.% Ta, which is smaller than the minimum distance below which the dislocation is instantaneously pinned by the Ta nanocluster at 300 K and also suggests a smaller dislocation line length of ~8 nm. In other words, the distance available for the dislocation to travel in NC-Cu-10 at.% Ta at room temperature is insufficient for the Cu lattice to influence the required external stress for deformation. This means that overcoming thermal barriers is the primary mode of deformation arising from externally applied stress. In terms of the flow-stress upturn, this shows that the dislocation cannot accelerate to a steady-state velocity at which phonon drag can influence the flow stress of the material as the dislocation travels freely through the Cu lattice between barriers without the addition of external loads. However, as temperature increases (particularly at 1073 K), the apparent distance between dislocations and nanoclusters can increase due to reduction in the misfit strains such that the spacing between dislocation barriers can exceed the critical *S* at which dislocations become pinned. Also, with increasing temperature, the drag coefficient and dislocation velocity also increase. For instance, at 1100 K, the absolute value of *B*_ph_ is about 8 orders of magnitude higher than at 298 K, meaning that at high temperatures, the density of phonons is increased such that phonon-drag forces play a larger role on mobile dislocations^25^. To determine the influence of temperature on the phonon density of states (PDOS), more atomistic simulations are required.

We know that the constitutive relation governing the change in flow stress due to phonon-drag effects is (ref. ^26^ and see Methods)

3 $$\tau _{{\mathrm{ph}}} = \frac{{B_{{\mathrm{ph}}}v}}{b},$$

where *v* is the terminal dislocation velocity, *b* is the Burger’s vector, and *B*_ph_ is a drag coefficient. In general, the absolute value of *B*_ph_ has been reported to be 1.6 × 10^−5^ Pa s at 298 K^25^ for pure Cu. Therefore, with a Burger’s vector of 2.56 Å, the *τ*_ph_ is approximately 97 MPa, which is significant when compared to the pure polycrystalline-Cu flow stress (~175 MPa^9^). Now from the perspective of NC materials strength, this phonon resistance may be negligible. However, in the case of NC-Ni, the reported increase in the flow stress at 10^3^ s^−1^ is about two times the flow stress at 10^−3^ s^−1^[8]. This increase can be caused by a few factors including grain growth, but the rise in flow stress in NC-Ni is more than 1 GPa indicating that the drag coefficient must be significantly higher for NC as compared to coarse-grained materials (similar to high-temperature effects). In fact, atomistic simulation of dislocation velocity results in Fig. 4b indicates that addition of Ta to pure Cu increases the drag coefficient (inverse of the linear segment slope) and Fig. 4c shows the density of long-wave acoustic phonons in Cu–Ta is higher than in pure Cu (indicative of more drag resistance). In particular, the presence of Ta in Cu induces disorder in the system and a shift of the acoustic modes toward lower frequencies is observed as Ta is introduced in Cu (Fig. 4c). This could lead to an increase in the phonon drag on a dislocation as compared to the case of pure Cu. A small shift of phonon modes toward lower frequencies with increase in temperature is typical of the thermal expansion and anharmonicity^27^. Indeed, the increased volume fraction of interfaces/defects in NC metals is the origin of their significantly altered thermal transport properties due to increase in phonon scattering^28^. Following Fig. 4b, the drag coefficient is inversely proportional to the slope of the linear region of the curve. Thus, the drag coefficient increases with an addition of Ta, which is in agreement with the calculated local PDOS (Fig. 4c). However, in the NC-Cu–Ta at 300 K, the dislocation gets pinned immediately after nucleation (velocity is zero), thus, negating any effects of phonons on dislocation motion. Therefore, the unique strain-rate-insensitive response in a stable NC material stems from the stability of the controlling microstructural length scale such as the grain size and Ta nanocluster size/spacing, which act as barriers that pin/slowdown defect propagation, scatter, and absorb phonons.

## Discussion

**Fig. 5:**
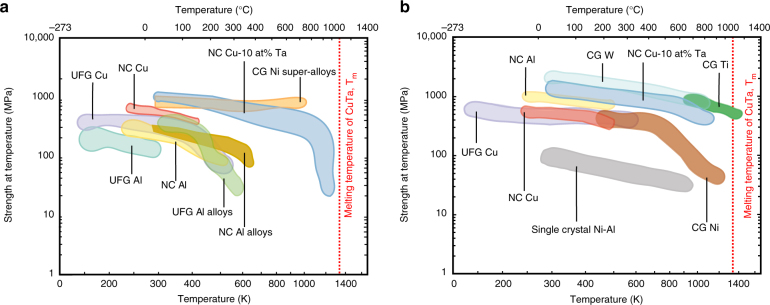
Strength at temperature vs. applied temperature for NC and UFG metals and alloys. Yield strength and flow strength for NC-Cu-10 at.% Ta’s data along with NC and UFG data reported in the literature at **a** quasi-static and **b** high strain rates. The rate of change of strength with temperature is lower for NC-Cu-10 at.% Ta as compared to other NC materials reported. Literature data are from ref. ^39^

The mechanical properties of a new class of NC material are explored. The results show that the high density of barriers to mechanical deformation through dislocation motion place NC materials among the strongest, most resilient, and most thermally stable known structural materials. When the microstructure of a NC material is stabilized, as in the case of NC-Cu-10 at.% Ta, the strength remains high despite harsh environmental conditions. For example, NC-Cu-10 at.% Ta is compared to other NC, ultra-fine-grained (UFG), and coarse-grained material systems in Fig. 5 through an Ashby plot of strength vs. temperature. Figure 5a corresponds to a quasi-static strain-rate range of 0.001–1 s^−1^ and Fig. 5b to a dynamic range of 1–10,000 s^−1^. The upper and lower bounds of the individual contours correspond to the stress response within the strain-rate limits over the given temperature range. For instance, the strength of the NC-Cu-10 at.% Ta alloy at 473 K was found to be 650 and 925 MPa for the strain rates of 0.001 and 1 s^−1^, respectively. Thus, a significant fraction of the room temperature strength (59% and 76%, respectively) was retained at this temperature indicating that a stable NC alloy can exhibit higher strength at high temperatures than its coarse-grained counterpart. Post-deformation imaging indicates that dislocations generated at quasi-static strain rates and at high strain rates combined with high temperatures are pinned by the Ta nanoclusters present within the grains. Similarly, at room temperature and high strain rate, the partial dislocations are also pinned by the same Ta nanoclusters. This combined with the atomistic results for the strengthening contribution of dislocation interaction with nanoclusters shows that the Ta nanoclusters are crucial to the strengthening of this material. As the material is stable such that the nanocluster dispersion along with the grain size remains similar even at temperatures up to 1073 K, the strength of the alloy remains high under such an extreme environment.

The Ta nanoclusters, which provide the stability of the NC structure are also shown to be the cause for the lack of flow stress upturn in NC-Cu-10 at.% Ta when tested at room temperature at strain rates exceeding 10^4^ s^−1^. The atomistic simulations show that with the reported average distance between nanoclusters <6 nm, the dislocation does not have sufficient room to reach its maximum velocity. This means that dislocation motion is predominantly motivated by the thermal activation mechanisms required for overcoming barriers rather than a need to overcome drag forces brought about by interactions with phonons. As a result, no upturn in flow stress is observed at strain rates up to 10^5^ s^−1^. Further, instead of full dislocation propagation through the matrix, the formation of partial dislocations is observed at room temperature and high strain rate. While mobile partial dislocations are also susceptible to phonon interactions at high strain rates, in the post-deformed microstructure observed here, twin density was not significantly changed when the strain rate increased by two orders of magnitude, i.e., the average measured twin area fraction through TEM images was found to only be about 4.0% and 5.3% for 10^3^ and 10^5^ s^−1^, respectively. Further, the twin widths were found to be much narrower than twins formed in pure Cu under similar testing conditions. The twin boundaries were found to be pinned by Ta nanoclusters indicating that even the partial dislocations, which are preferred at high strain rates and low temperatures, do not have sufficient space to accelerate to a velocity at which interaction with phonons plays a major role.

As testing temperature is increased, however, an upturn in flow stress can be observed though the distance between Ta nanoclusters remains low. As temperatures increase, the activation energy for nucleating dislocations also increases. The bright-field scanning transmission electron microscopy (BF-STEM) images of Fig. 3d reveal a high number of dislocations in a sample tested at high strain rate combined with high temperature as compared to the limited number of full dislocations generated within the sample tested at room temperature and high strain rate (Fig. 3c). Further, with elevated temperature, a shift in the PDOS curve, as shown by the atomistic simulations of Fig. 4, indicates that a higher phonon density exists resulting in a higher drag coefficient. The combination of increased energy for generating and propagating dislocations with an increase in phonon-drag coefficient requires a significantly greater stress than that required at quasi-static states to further deformation. Hence, an upturn in normalized flow stress is observed at lower strain rates as testing temperature is increased.

Therefore, even under intense conditions, this alloy still demonstrates a remarkable thermal-mechanical stability wherein the yield and flow stresses remain significantly higher than almost all NC and UFG alloys reported in literature. Further, this discovery points to a unique materials design principle through which materials with extraordinary strength (approximately one-half the theoretical limit) and high-temperature stability can be developed for advanced technological applications. Hence, there is potential in designing new, tougher materials for advanced applications, such as those from power generation-reliant industries, low-temperature (cryogenic) applications, and space shielding (unique spall-fragmentation behavior). Overall, we have shown, that the flow-stress behavior in stable NC materials diverges from the conventional understanding, and the strain rate that marks the onset of the transition to the phonon-drag regime can, at the very least, be delayed to higher strain rates, thus providing a clear path for designing materials for low temperature as well as extreme strain-rate applications.

In summary, the results obtained in this work point to the following observations: microstructural features, such as grain size and Ta nanoclusters are relatively insensitive to dynamic mechanical deformation at elevated temperatures, i.e., grain and Ta nanocluster coarsening is negligible under tested conditions; as a direct result of the inherent and extremely high density of interfaces (Ta-phase and Cu grain boundaries), NC-Cu-10 at.% Ta was shown to exhibit an increased phonon-drag coefficient relative to coarse-grained pure copper; the short distance between the Ta nanoclusters contributes (limiting condition) to a stable flow-stress response with increasing strain rate as both full and partial dislocations are prevented from attaining steady-state velocities at which drag forces from phonon-dislocation interactions become dominant; while the grain and phase-boundary densities are high in NC-Cu-10 at.% Ta, the average deformation induced twin area fraction was marginal, ~5% at the highest strain rates, i.e., 10^3^ and 10^5^ s^−1^ indicating that twins play a minimal role if any on altering dislocation physics; despite having an elevated drag coefficient, NC-Cu-10 at.% Ta exhibits rate-independent behavior at room temperature due to dislocations becoming pinned immediately after nucleation (velocity is zero); a limited dislocation velocity is a major factor in observing the delay in flow-stress upturn in NC-Cu-10 at.% Ta at higher strain rates; increasing temperature results in a decrease in the activation energy required for dislocations to overcome barriers (i.e., decreased dislocation wait time and increased dislocation velocity) as well as an increase in the phonon density (i.e., increased drag coefficient)—both of which contribute to a sharp rise in the flow stress with increasing strain rate at temperatures above 473 K in NC-Cu-10 at.% Ta; and contrary to typical NC materials, the strain- rate sensitivity remains low with an activation volume of ~50*b*^[3] 22^.

## Methods

### Powder processing and consolidation

NC-Cu-10 at.% Ta powders were processed utilizing high-energy cryogenic mechanical alloying with elemental Cu and Ta powders (−325 mesh and 99.9% purity). Details of the ball milling process can be found in Supplementary Note 1. The NC-Cu-10 at.% Ta powder was consolidated to bulk via equal-channel angular extrusion (ECAE) through an angle of 90° at 700 °C. Four passes were completed following route B_*c*_ in order to obtain an equiaxed nanostructure. More details on the powder consolidation can be found in Supplementary Note 1. Specimens for mechanical testing were then machined from the consolidated billets via wire electric discharge machining into 3 mm length by 3 mm diameter cylinders. Further details related to the processing and impurity levels can be found in refs. ^15,21,29,30^.

### Microstructural characterization

To obtain grain-size distributions and microstructural characteristics, HRTEM was employed. HRTEM characterizations were carried out in the as-received and post-deformed conditions using an aberration corrected ARM-200F at 200 KeV. Multiple bright-field and dark-field images were captured in both the HRTEM and STEM modes to assess the microstructure and quantify statistics such as grain-size distribution and so on. For HRTEM characterizations, samples were prepared through conventional thinning procedures as outlined in Supplementary Note 2. To investigate the active deformation mechanisms and the reasoning behind the high-temperature and strain-rate response in these alloys, ex situ microstructural characterization was employed using the same HRTEM techniques employed to investigate the as-received microstructure. Atom probe tomography (APT) was performed using a Cameca Local Electrode Atom Probe (LEAP) 5000 XS instrument. The specimens were lifted out from the sample, which had been ECAE-processed at 700 °C. Details regarding APT specimen preparation can also be found in Supplementary Note 2. During the APT experiments, the specimens were maintained at a base temperature of 45 K (−228 °C) and run in the pulsed laser mode with a pulse rate of 333 kHz, pulse energy of 50 pJ, and a target evaporation rate of 0.5%. The APT data was reconstructed using Cameca’s Integrated Visualization and Analysis Software (IVAS) 3.6.12. Further, the twin area fraction was calculated by considering the total area of grains containing twins divided by the total area within the TEM micrographs used.

### Mechanical characterization at quasi-static conditions

Quasi-static compression tests of specimens over a temperature range from ambient up to 1073 K (800 °C), were performed using an Instron load frame equipped with a 50 kN load cell and an ATS clam-shell heating furnace capable of a maximum temperature of 1473 K (1200 °C) in lab air. The specimens for compression were cylinders 3 mm in diameter and length (aspect ratio 1.0). Compression tests were conducted at 223, 298, 473, 573, 873, and 1073 K, with strain rates ranging from 10^−2^ to 1 s^−1^. More details pertaining to quasi-static testing can be found in Supplementary Note 3. Scanning electron images confirmed that a negligible oxide film (<10 μm in thickness) was formed on the surface of the cylinders as a result of exposure to the elevated temperatures, as measured post testing.

### Mechanical characterization at high-strain-rate conditions

High-strain-rate compression tests of specimens over a temperature range from ambient up to 1073 K were performed using an Inconel 718 Kolsky bar with 12.7 mm diameter and a tube furnace capable of reaching a maximum temperature of 1173 K. The specimens for compression high-strain-rate testing were also cylinders 3 mm in diameter and length (aspect ratio 1.0). Tests were conducted at various temperatures ranging from 298 to 1073 K with strain rates ranging from 1 × 10^3^ to 2 × 10^4^ s^−1^. Details of Kolsky bar testing are found in Supplementary Note 4 and high-rate data analyses in Supplementary Note 10. At high strain rates and high strain levels, adiabatic heating causes softening in samples, so flow stress should be determined at low levels of strain, see Supplementary Figure 2. To this end, flow stress is taken at a 10% strain level and the rise in temperature resulting from adiabatic heating as determined by the method of Kapoor and Nemat-Nasser^31^ was calculated. Assuming 100% of the work in the sample is converted to heat at the 10% strain level, adiabatic heating causes less than an ~40 K rise in temperature at testing temperatures below 473 K and <~20 K for testing temperatures above 473 K.

### Taylor anvil experiment

As no upturn in flow stress was observed in the NC-Cu-10 at.% Ta alloy up to strain rates of 10^4^ s^−1^, a Taylor anvil experiment capable of obtaining strain rates on the order of 10^5^ s^−1^ was conducted by directly firing a 3 mm diameter ×8 mm-long cylindrical specimen using a compressed nitrogen gas gun at a C350 maraging steel, rigid target plate. Further details regarding the Taylor impact test can be found in Supplementary Note 5. The initial and post-deformed lengths of the projectile can be used to approximate the yield stress of the projectile material using

4 $$\sigma _{\mathrm{y}} = \frac{{ - \rho v_{\mathrm{s} }{\,} f\left[ {l_0\left( {l_1 - X} \right)} \right]}}{{\left( {l_0 - X} \right)\left( {l_0 - l_1} \right)}},$$

where *ρ* and *v*_s_ are the density and velocity of the projectile, respectively, *f* is a factor dependent on the rate of deceleration of the projectile (0 < *f* < 1), where *f* = 0.5 corresponds to linear deceleration, *l*_1_ and *l*_0_ are the deformed and initial specimen lengths, respectively, and *X* is the length of the undeformed section post testing^16^. By assuming that Cu is the primary source for deformation mechanisms in NC-Cu-10 at.% Ta, the fitting factor *f* used here is 0.64, which is the average fitting factor used by House et al.^16^ in their measurements on high-purity Cu. The strain rate of deformation is estimated assuming uniform deceleration by

5 $$\dot \varepsilon = \frac{{v_{\mathrm{s}}}}{{2l_{\mathrm{d}}}},$$

where *l*_d_ is the length of the deformed section of the sample^32^. The yield stress determined from Taylor anvil experimenting is merely an estimate and finite element simulations are needed to validate the experimental values of the flow stress at strain rates above 10^4^ s^−1^^33^. To this end, finite element simulations based on a Johnson-Cook model^34^. Details regarding the finite element model are given in Supplementary Note 5. With the finite element model accurately predicting the overall geometry of the post-deformed experimental profile, the resultant flow stress is taken and indicated by the red mark in Fig. 1, where the maximum strain rate measured one element from the impact surface is ~2 × 10^5^ s^−1^ s^−1^. This confirms the Taylor anvil experiment results showing that no significant upturn in flow stress occurs at strain rates up to 10^5^ s^−1^.

### Atomistic simulations of dislocation velocity

Molecular-dynamics simulations were employed to investigate the effect of tantalum particles on the velocity of an edge dislocation in NC copper. Atomistic simulations were performed for both the Cu–Ta and pure Cu systems using angular-dependent potential (ADP), which was developed by Pun et al.^35^ This ADP interatomic potential was parameterized using an extensive database of energies and configurations from density functional theory calculations of energy differences between various crystal structures of pure Cu and pure Ta, the formation energies of coherent Cu–Ta interfaces, and the binding energies of several ordered compounds^35^. An edge dislocation was introduced in a box of copper atoms as described by Bhatia et al.^36^ Subsequently, periodic boundary conditions were prescribed along the *X*-direction (Burger’s vector direction) and *Z*-direction (dislocation line direction); while, a free boundary condition was maintained along the *Y*-direction (slip plane normal). Initially, the edge dislocation was created with three different computational cell sizes 100*b* × 100*b* × 80*b*, 160*b* × 160*b* × 80*b*, and 200*b* × 200*b* × 80*b*, where *b* is the Burgers vector (*b* = 2.556 Å). The simulation cell size of 160*b* × 160*b* × 80*b* with ~2.5 million atoms was chosen based on the convergence studies such that the simulation cell is sufficiently large to assess the steady-state dislocation velocity. Further, the edge dislocation dissociated into two partials with a stacking fault width of 3.5 nm after the conjugate-gradient relaxation for 1 ns, which is found to be in good agreement with the reported literature values of 3.0–3.6 nm for copper^37^. A tantalum nanocluster was introduced in a copper nanocrystal away from the dislocation and the atomistic model was equilibrated using a canonical ensemble. The top and bottom regions (~10 Å) along the *Y*-direction were fixed and a constant shear stress was applied to the top of the simulation box in the *X*-direction to obtain the steady-state dislocation velocity.

### Atomistic simulations of phonon density of states

The high density of barriers, such as grain/twin boundaries and the high number density (6.5 × 10^23^ m^−3^) of nanoclusters (Fig. 3) can lead to scattering, absorption, and transmission, which should distort the local PDOS and produce perturbations in the elastic/plastic front as shown in Fig. 4. Thus, we analyzed the effects of tantalum on the PDOS of Cu to provide further insight into the physics governing the anomalous mechanical behavior observed in NC-Cu-10 at.% Ta. Atomistic calculations of PDOS were performed by the method developed by Kong^38^ and implemented in large-scale atomic/molecular massively parallel simulator (LAMMPS). The PDOS for Cu at 300 K was found to be in good agreement with the reported experimental and theoretical measurements^38^. The presence of Ta in copper induces disorder in the system and a shift of the acoustic modes toward lower frequencies is observed as Ta is introduced in Cu (Fig. 4c). This could lead to an increase in the phonon drag on a dislocation as compared to the case of pure Cu. A small shift of phonon modes toward lower frequencies with increase in temperature is typical of the thermal expansion and anharmonicity^27^.

### Data availability

The data that support the findings are available from the corresponding authors upon request.

## Electronic supplementary material

Supplementary Information
