# Supplementary material for: Anomalous mechanical behavior of nanocrystalline binary alloys under extreme conditions
Source: Nat Commun. 2018 Jul 12;9:2699. doi: 10.1038/s41467-018-05027-5 (PMC6043485; doi:10.1038/s41467-018-05027-5)
Supplement: Supplementary file 1 — Supplementary Information [file 41467_2018_5027_MOESM1_ESM.pdf]

Supplemental Information

Anomalous mechanical behavior of nanocrystalline binary alloys under extreme conditions

Turnage et al.

Supplementary Figures 1-10
Supplementary Table 1

Supplementary Note 1: Powder processing and equal channel angular extrusion (ECAE)

Nanocrystalline (NC) Cu-10at.%Ta powders were processed utilizing high-energy cryogenic mechanical alloying. Elemental Cu and Ta powders (-325 mesh and 99.9% purity) were loaded into a hardened steel vial in the appropriate proportion along with the milling media (440C stainless steel balls) inside a glove box with an Ar atmosphere (oxygen and H_2O are < 1ppm). The vials were loaded with 10 g of the Cu-Ta powder as well as the appropriate amount of media to ensure a ball-to-powder ratio of 5-to-1 by weight. A SPEX 8000 M shaker mill was utilized to perform the milling at cryogenic temperature (verified to be ~ -196 °C) for 4 hrs using liquid nitrogen. The NC-Cu-10at.%Ta powder was consolidated to bulk via equal channel angular extrusion (ECAE). Before starting the ECAE process, the die assembly used for processing the billets was preheated to 623 K (350 °C) to minimize thermal loss during the ECAE processing. The billets, heated and equilibrated to 973 K (700 °C) for 40 min, were dropped into the ECAE tooling as quickly as possible from the furnace and extruded at a rate of 25.5 mm/s. These steps were repeated 4 times following route B_c [1–3] to prevent imparting a texture to the consolidated powder. By extruding through an angle of 90°, a total strain of 460% was imparted onto the powder-containing billet as a result of processing.

Supplementary Note 2: Microstructural characterization

Specimens for TEM characterization were prepared through conventional thinning procedures where a 3 mm disk from the bulk specimen was thinned to about 70 μm following which the specimens were dimpled to about a 5 μm thickness. Ion milling was performed using a Gatan Precision Ion Polishing System (PIPS) under liquid nitrogen temperatures to obtain electron-transparent regions in the specimens. The samples were also plasma cleaned in Ar prior to TEM observations to reduce contamination.

The specimens for APT were lifted-out from the sample which had been ECAE processed at 700 °C and placed on a pre-fabricated Si post using an FEI Nova 600 NanoLab dual-beam scanning electron microscope and focused ion beam (SEM/FIB) equipped with an Omniprobe micromanipulation system. Once welded to the Si post, each specimen was annular milled to a final tip diameter of 60-100 nm.

Supplementary Note 3: Mechanical characterization at quasi-static conditions

The samples for quasi-static testing were held at the testing temperature for 30-minutes prior to loading to provide uniform temperature within the specimen. The push rods of the load frame were constructed of Inconel 718. Polished WC-disks lubricated with graphite were used as platens for compression testing. A thermocouple embedded in the Inconel rod was used to measure the temperature of the specimen with the assumption that the Inconel rod and specimen would reach thermal equilibrium within the 30-minute interval.

Supplementary Note 4: Mechanical characterization at high-strain rate conditions

As illustrated in Supplementary Figure 1, the Kolsky bar uses three rods of the same diameter and material to measure the stress-strain response of a specimen. These are the striker bar,

incident bar, and transmitted bar. The striker bar impacts the incident bar generating a strain pulse that travels to the specimen, which is held in place between the incident and transmitted bars. When the incident pulse encounters the specimen, it splits into a reflected pulse and a transmitted pulse. The reflected pulse measured in the incident bar provides information on the strain in the specimen while the transmitted pulse measured in the transmitted bar provides information on the stress observed by the specimen. Further details of the Kolsky bar test method can be found in the work by Kale et al. [4]. The addition of temperature to the Kolsky bar can distort the 1D wave approximation by generating a gradient of elastic properties within the bar. Inconel 718 was used as the bar material to minimize the effects of temperature, and correction for the remaining thermal gradient has been applied using the work of Bacon et al. [5] in the form

$$\varepsilon = \frac{4c_T E(\varepsilon_i - \varepsilon_t)}{(E_T + \sqrt{E * E_T})l_s} \quad (1)$$

$$\sigma = \frac{\varepsilon_t E_T A_b}{A_s} \quad (2)$$

where ε and σ are the strain and stress in the sample, respectively, c_T is the wavespeed in the bar at the testing temperature, E and E_T are the elastic moduli of the bar at room temperature and testing temperature, respectively, ε_i and ε_t are the strain measured in the incident bar and transmitted bars, respectively, l_s is the sample length, and A_s and A_b are the cross-sectional areas of the sample and bar, respectively. As a result of the short deformation time during Kolsky bar testing, force equilibrium on each side of the specimen is not attained until plastic deformation has already begun.

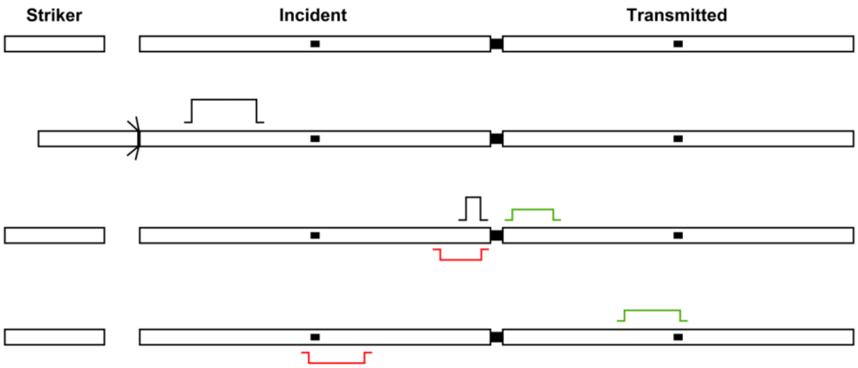

Supplementary Figure 1. Schematic showing wave propagation through a Kolsky bar. The black wave is the incident wave generated by impact of a striker bar with the incident bar. The incident wave travels down the bar to the specimen where the wave decomposes into transmitted (green) and reflected (red) waves.

Supplementary Note 5: Taylor anvil experiment for yield stress at 10^5 s^{-1}

A Taylor anvil experiment capable of obtaining strain rates on the order of 10^5 s^{-1} was conducted by directly firing a 3 mm diameter x 8 mm long cylindrical specimen using a compressed nitrogen gas gun at a C350 maraging steel, rigid target plate. The gas gun was powered by a

pressure tank filled to 240 psi with nitrogen gas. The sample was loaded directly into the 12.7 mm diameter x 1,500 mm long barrel and backed with a cotton plug placed directly behind the gas release valve. The sample was press fit into a polymer sabot, which high speed photography confirmed did not slide forward until after the initial impact. The velocity of the projectile was measured at the end of the gas gun barrel immediately before impact where the projectile breaks two wires in a circuit spaced 36 mm apart. The resultant drop in potential across the circuit is measured as a function of time, so the velocity of the projectile can be measured as the time between potential drops divided by the distance between the wires. The velocity for these experiments measured approximately 240 m/s. Finite element simulations based on a Johnson-Cook model [6] with parameters listed in Supplementary Table 1 are shown to result in a reasonable fit to the lower strain rate data for NC-Cu-10at.%Ta (see Supplementary Figure 2a).

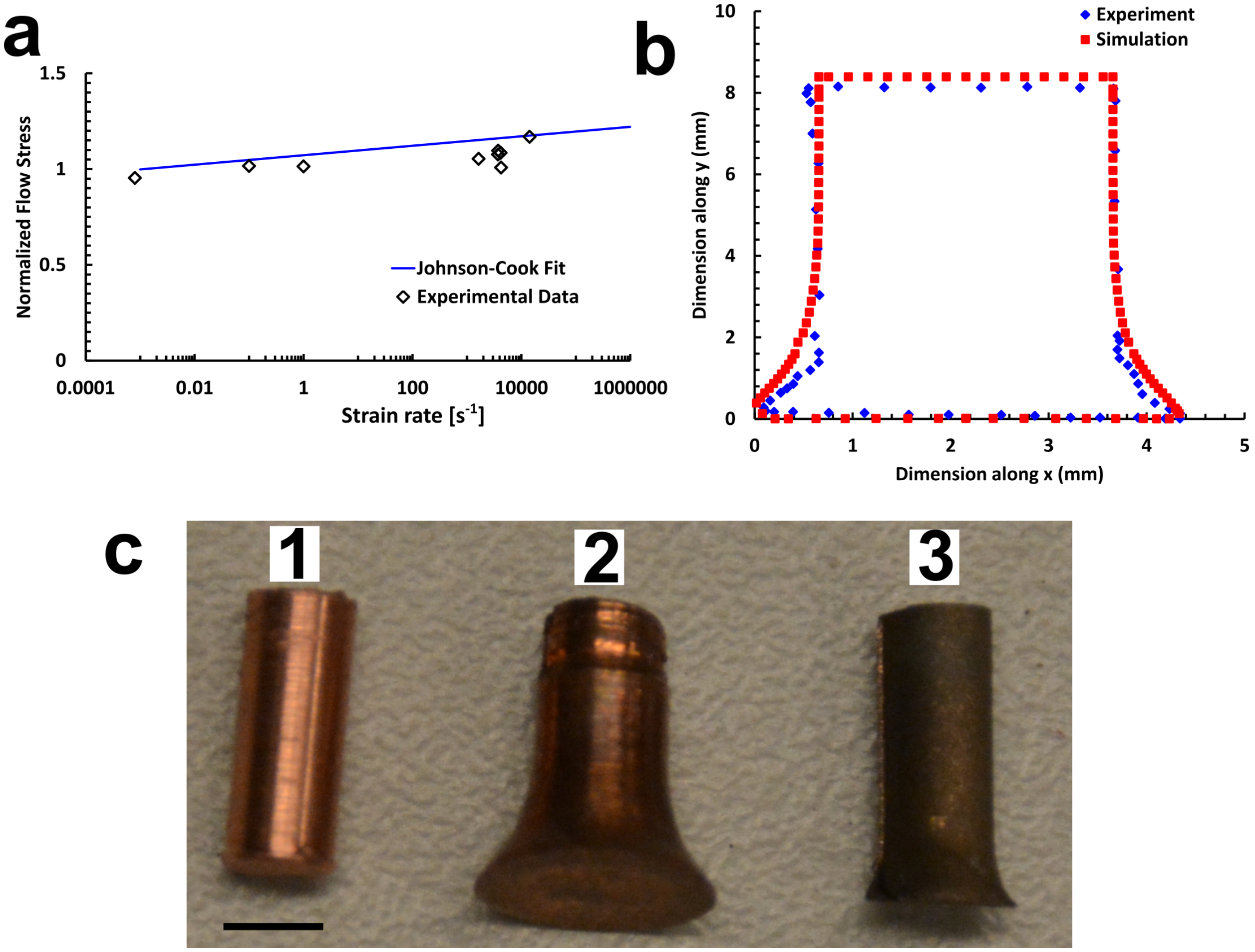

Supplementary Figure 2. Fits of model to Taylor anvil experiment data. a) The flow stress as a function of strain rate for the experimental data obtained from quasi-static and Kolsky bar testing compared to the Johnson-Cook model fit with parameters from Table S1. b) Deformed geometry from the simulation is shown to match reasonably well with experimental results. The maximum strain rate obtained from simulations one element away from the impact surface measured approximately 2×10^5 s^{-1}. c) Samples from Taylor anvil experiments, which show representative specimens of: 1) an undeformed, pure Cu specimen, 2) the large deformation of a

pure Cu specimen and 3) the relatively minimal deformation of a Cu-Ta sample. The black bar in (c) corresponds to a length of 3 mm.

Supplementary Table 1. Johnson-Cook Parameters for NC-Cu-10at.%Ta

Model parameters	
A	1,229 MPa
B	303 MPa
N	0.264
C	0.01
$\dot{\varepsilon}_0$	1 s^{-1}

Supplementary Note 6: Compressive mechanical response

The stress-strain responses are provided in Supplementary Figure 3. The compressive curves display an elastic- nearly perfectly plastic behavior over the entire temperature range with no significant strain hardening beyond 2.5% strain. Very little strain rate sensitivity is apparent at 298 K, and even with a testing temperature of 473 K, the flow stress of the nanocrystalline material is around 1 GPa. Interestingly, despite the thermal dependence at temperatures greater than 298 K, little rise in flow stress is observed as a result of decreasing the temperature to 223 K.

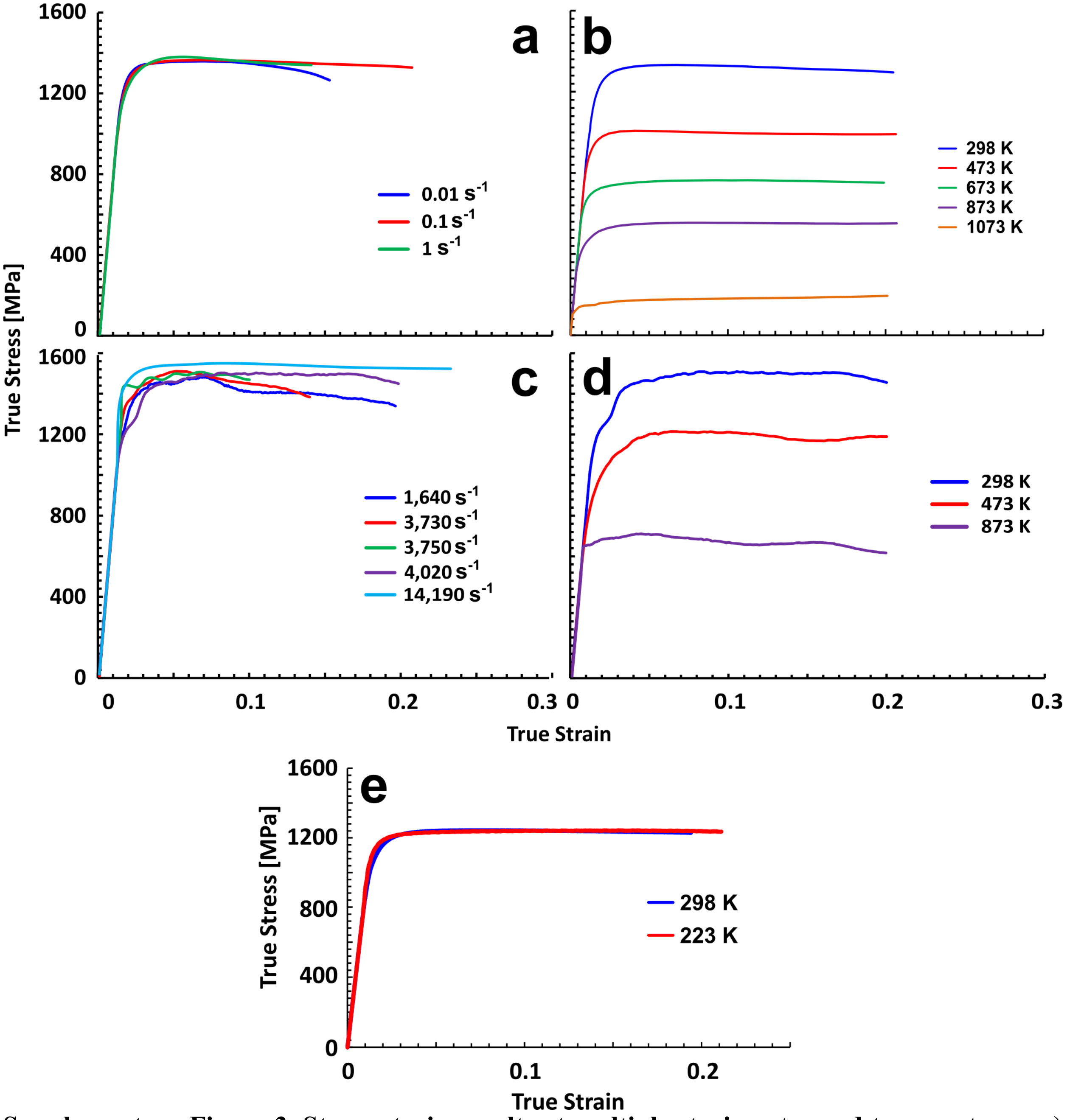

Supplementary Figure 3. Stress-strain results at multiple strain rates and temperatures. a) Quasi-static compression at room temperature, b) 10^{-2} s^{-1} compression for temperatures ranging from room temperature to 1073 K (800 ˚C), c) high strain rate compression at room temperature, d) 4×10^3 s^{-1} compression for temperatures ranging from room temperature (298 K) to 873 K (600 ˚C), and e) comparison of 10^{-3} s^{-1} compression at 298 K and 223 K for NC-Cu-10at.%Ta.

Flow stress values were taken for each strain rate and temperature at 10% plastic strain as mentioned in the main document, but to compare the effects of strain hardening on the flow stress upturn, a comparison of flow stress values taken from different strain levels can be seen in Supplementary Figure 4 for the room temperature tested NC-Cu-10at.%Ta, coarse grained Cu, and coarse grained Ta. Here, little difference in normalized flow stress can be seen as a result of strain level for NC-Cu-10at.%Ta.

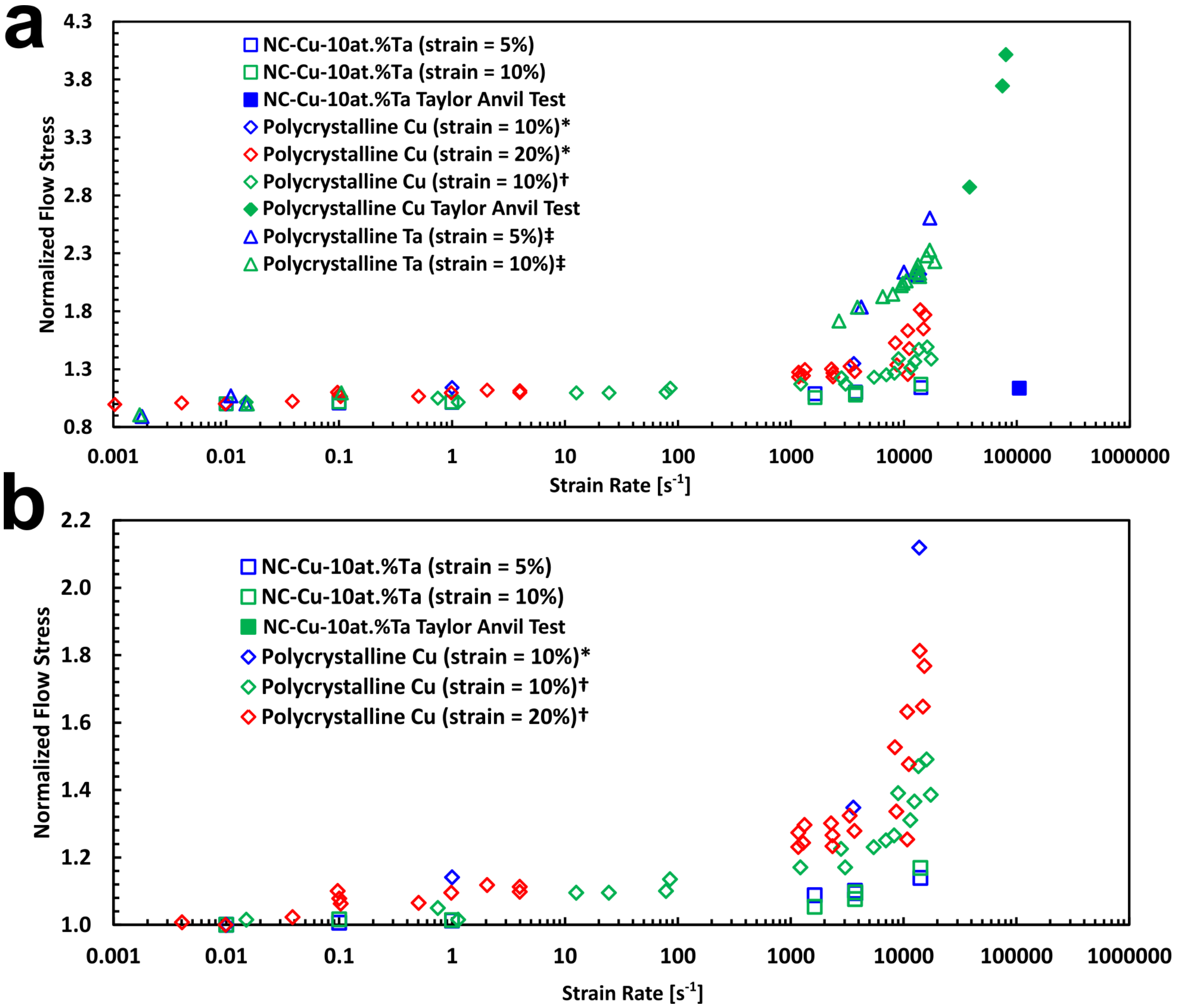

Supplementary Figure 4. Flow stress-strain rate variation as a function of strain level. (a) NC-Cu-10at.%Ta data taken at strains of 5 and 10% are compared with polycrystalline Cu from Jordan et al. [7](*) at 10 and 20% strain as well as from Follansbee and Kocks [8] (†) at 10% strain. Also, polycrystalline Ta from Rittel et al. [9] (‡) at 5 and 10% strain. Limited variation is observed in normalized flow stress at each of the aforementioned strain levels. The NC-Cu-10at.%Ta data remains lower than the polycrystalline data and no upturn is observed in the data presented here. Numerical analysis following the methods of [10] will be required to more accurately determine the appropriate yield stress. (b) By removing the Ta and all Taylor anvil experiment data, the flow stress upturn in polycrystalline Cu is more clearly seen to occur at a lower strain rate than that of the stabilized NC-Cu-10at.%Ta.

Supplementary Note 7: As-received microstructure

Primary microstructural characterization using TEM revealed the presence of nanocrystalline grain sizes for the copper and tantalum particle phases with an average grain size of 50 ±18 nm for Cu. Tantalum particles are shown to exhibit a range of sizes from atomic sized clusters ($d < 14$ nm) to larger particles ($d > 14$ nm). The larger particles and atomic sized clusters have an average diameter of 32 ± 8 nm and 3.2 ± 0.9 nm, respectively. The size distributions were obtained from areas similar to that seen in the Extended Data Figure 2 of [11], and were averaged over 300 grains. It is important to note that TEM images show all material through the thickness of the specimen such that 3-dimensional data is projected onto a 2-dimensional image. To account for any error from this form of measurement, Monte Carlo simulations of the powder packing process where performed which show that the 2-dimensional and 3-dimensional measurements are comparable and consistent with an earlier 3D atom probe study [12]. Further, it is noted that the microstructure has a few twins, but the formation of nano-twins during processing for this composition is minimized due to the presence of fine nanoclusters [13].

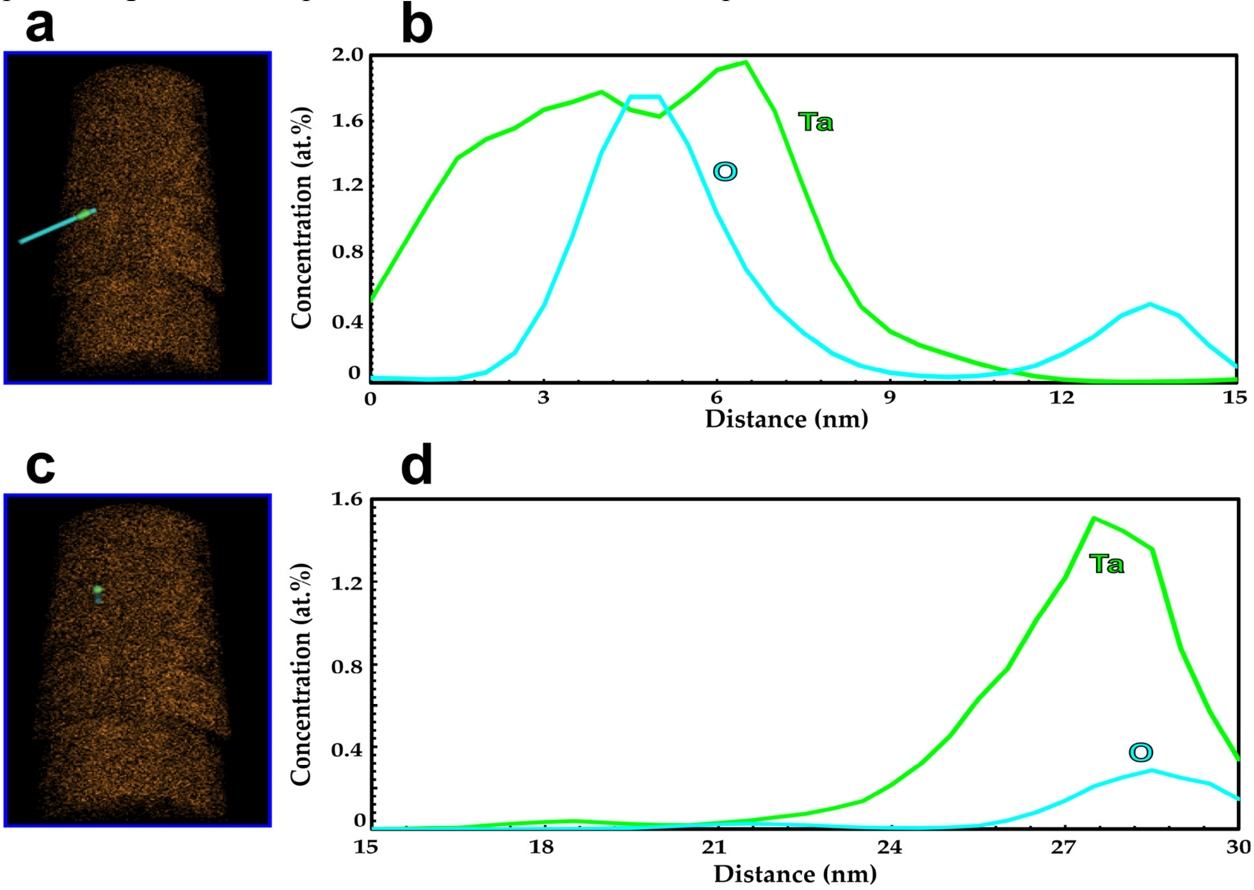

Supplementary Figure 5. As-received microstructural characterization. a) and c) Atom probe reconstruction showing Cu ions, a Ta particle (delineated with a 7.28 at.% TaO isoconcentration surface), and an analysis cylinder placed within two different Ta particles b) and d) 1-dimensional concentration profile generated from the analysis cylinder showing the O and Ta concentration within the particle of a) and c). The profile in b) indicates a core-shell structure with O concentration highest in the core and Ta concentration highest in the shell.

Supplementary Figure 5 shows the 3-dimensional reconstructed tip of NC-Cu-10 at% Ta. The orange spheres are the Cu ions of the matrix material, while the green surfaces identify and mark the large particles of Ta present within the alloy system. (These regions are delineated from the matrix of the alloy using a TaO isoconcentration surface set to 7.28 at% TaO, where the interior of the surface has a higher concentration of Ta and O compared to the lower content outside the surface in the Cu matrix). Two of these smaller isoconcentration surfaces had 1-dimensional concentration profiles run through them to measure the partitioning behavior of Cu, Ta, and O within them. This yielded some interesting results that correlate nicely with the variety of Ta particles present in the TEM micrographs shown in Figure 3. One of the 1-dimensional profiles indicates that the particle has a core-shell structure. This is seen by the O composition reaching a maximum where the Ta composition decreases and the Ta composition reaching its maximum on both sides of this O peak. This signifies an outer shell that is enriched in Ta, and as the core is reached, the Ta content decreases as an increase in O occurs. This particle is ~ 5 nm in diameter. When performing the same analysis on a slightly smaller (~2-4 nm) surface, the O and Ta composition reach their maximum compositions at the same point. This indicates the core-shell structure is not present in this particle, which corresponds to the variety of particle structures seen in the TEM micrographs with smaller particles displaying a more constant contrast across themselves indicative of a uniform composition and large particles having a changing contrast due to a chemical compositional change. The final figure is an image taken from the interior of a smaller particle showing the clustering of Ta and TaO particles within the interior of the isoconcentration surface compared to the surrounding matrix of Cu ions.

Supplementary Note 8: Post deformed microstructure

Post deformation nanostructure imaging was performed using TEM and STEM imaging following the same methods utilized for the as received microstructure. From the TEM observations presented here (Supplementary Figure 6) on NC-Cu-10at.%Ta under high strain rate conditions, nucleation of partial dislocations is favored at RT due to the high applied stress which surpasses the barrier for twinning based deformation. This can also be identified from the micrographs indicated in Figure 3 where twins having narrow widths can be identified. The growth of the twins is restricted due to the presence of Ta nanoclusters in NC-Cu-10at.%Ta [13], so the stability of the nanostructure is maintained. However, at elevated temperatures, the deformation shifts from twin based to dislocation based where, again, the thermal energy favors dislocation slip. An appreciable dislocation density can be identified for high temperature testing conditions implying an absence of dislocation absorption by neighboring grain boundaries. In the case of conventional nanocrystalline materials, grain boundaries act as a source for dislocation generation, and once the dislocations are free to traverse the grain, they will be absorbed at the opposite grain boundary which acts as a sink unless, as in the case of NC-Cu-10at.%Ta, the dislocations are pinned by defects such as Ta nanoclusters. Figure 2 in the main paper illustrates the variation in inter-cluster spacing for Ta nanoclusters as a function of strain rate at various testing temperatures. It can be seen that both temperature and deformation rate have minimal influence on the spacing, which confirms the stability of the alloys up to 1073 K. The restricted motion of dislocations is the result of an un-pinning stress that the dislocation must overcome for motion to resume. The propensity of dislocation slip increases for both quasi-static and dynamic testing conditions with an increase in testing temperature, which helps in overcoming the energy barrier required for dislocation based activity in these systems. However, the mean free path for

the dislocation motion is dependent on the grain size and density of the nanoclusters that are embedded in the material system. In other words, the increased mobility of dislocations lowers the flow stress of the material as temperature increases, but the stable density of Ta nanoclusters allows the material to retain high strength at temperature relative to pure NC and CG Cu.

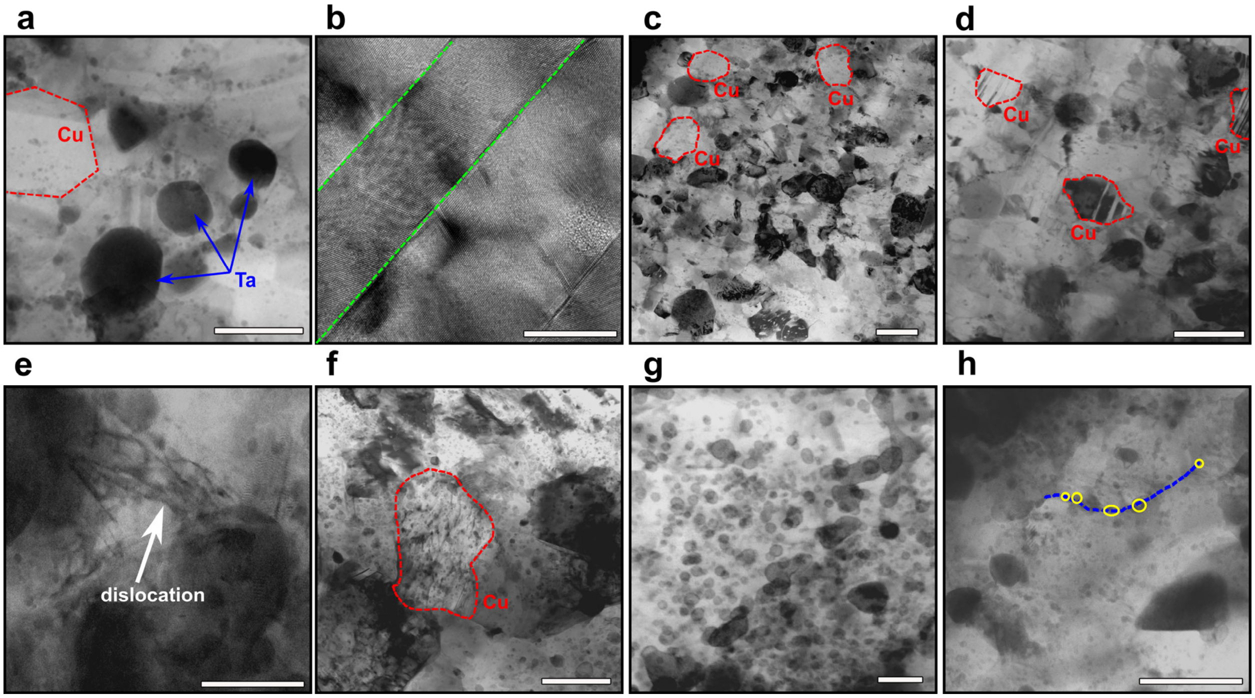

Supplementary Figure 6. TEM microstructure characterization of NC-Cu-10at.%Ta under as-received and multiple post-deformed conditions. (a) As-received microstructure of NC-Cu-10at.%Ta ECAE processed at 973 K. (b) HRTEM micrographs of twins in the as-received microstructure. (c) and (d) Post-deformed micrograph of Taylor anvil tested sample. Very small area fraction of Cu grains having twins can be seen even at strain rates in the order of 10^5 s^{-1}. (e) Post-deformed micrograph of sample tested at 873 K and a strain rate of 4 x 10^3 s^{-1}. Emission of dislocations evident at higher temperature and strain rates. (f) and (g) TEM BF images of quasi-static (0.01 s^{-1}) tested sample at 1073 K showing distribution of the Ta nanoclusters. (h) TEM BF image of quasi-static (0.01 s^{-1}) tested sample at 473 K showing dislocation interacting with particles. The whit scale bar in a, c-d, f and g correspond to a length scale of 100 nm, in b it corresponds to 10 nm, in e it corresponds to 50 nm and in g it corresponds to 20 nm.

Supplementary Note 9: Ta concentration effects on Hall-Petch strengthening

The addition of Ta influences the stability of the nanostructure of the Cu-Ta alloy system. As a result, very fine nanocrystalline grain sizes result from the high temperature processing discussed above. In order to determine the influence of Ta concentration on the strengthening obtained by this grain refinement, i.e., Hall-Petch behavior, hardness as a function of grain size for various NC-Cu results from literature are presented in Supplementary Figure 7 compared with NC-Cu-xat.%Ta (x being 1,3,5 and 10). From the plot it is evident that the NC-Cu-10at.%Ta continues to show Hall-Petch hardening (no inverse Hall-Petch) even up to a grain size of 6 nm (point with '♦' marker in Supplementary Figure 7).

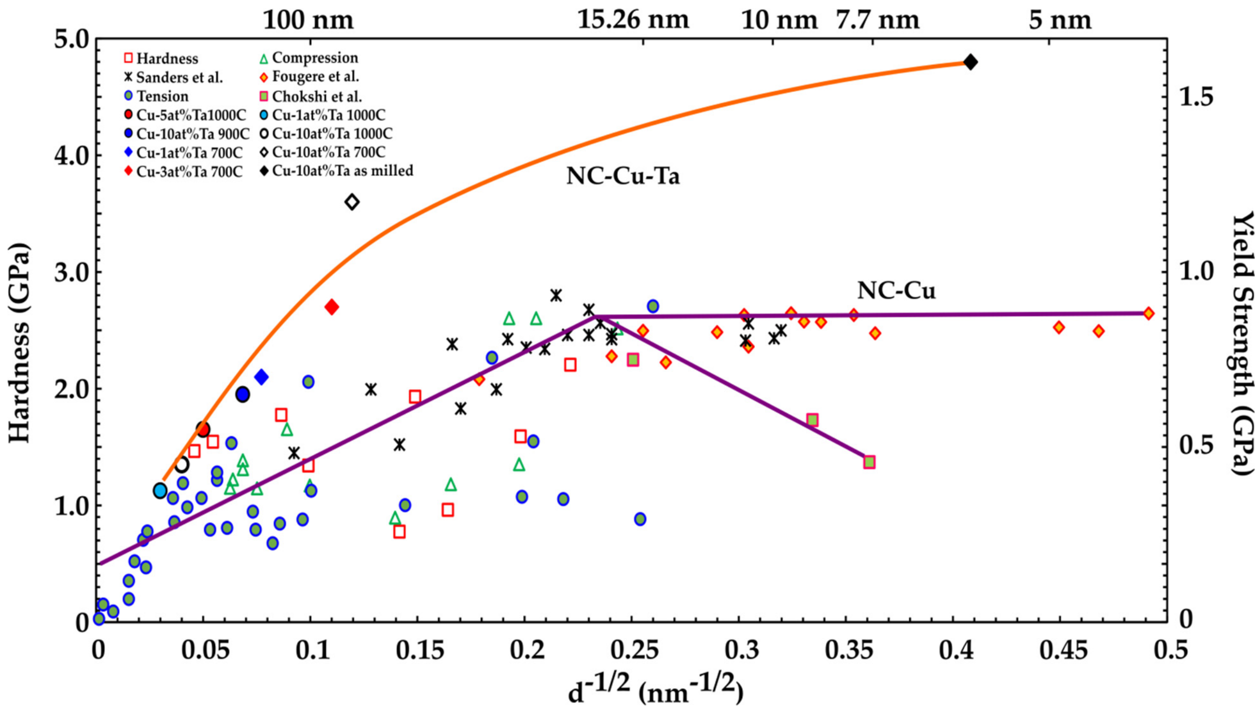

Supplementary Figure 7. Hardness and yield strength (compression and tension) as a function of grain size for multiple Cu compared with Cu-x at.%Ta (x being 1,3,5 and 10). Hardness values obtained from literature shows the hardening behavior is almost constant for very fine grain sizes (< 10 nm). The only exception to this behavior is that shown by Chokshi et al. [14] where inverse Hall-Petch effect has been seen in NC-Cu. However, NC-Cu-10at.%Ta is shown to follow the Hall-Petch hardening almost up to a grain size of 6 nm. Hardness values were obtained from [14–19], compression yield strength values were obtained from [16,18,20–23] and tensile yield strength values were obtained from [18–20,23–28].

Supplementary Note 10: High-rate data analyses

Supplementary Figure 8 represents a comparison of the flow stresses of various high purity Cu and Ta materials representing differing grain sizes, and it can be clearly seen that the flow stress of the NC-Cu-10at.%Ta is higher than all other materials shown. This figure is the same as seen in Figure 1A of the main document only with non-normalized values of flow stress.

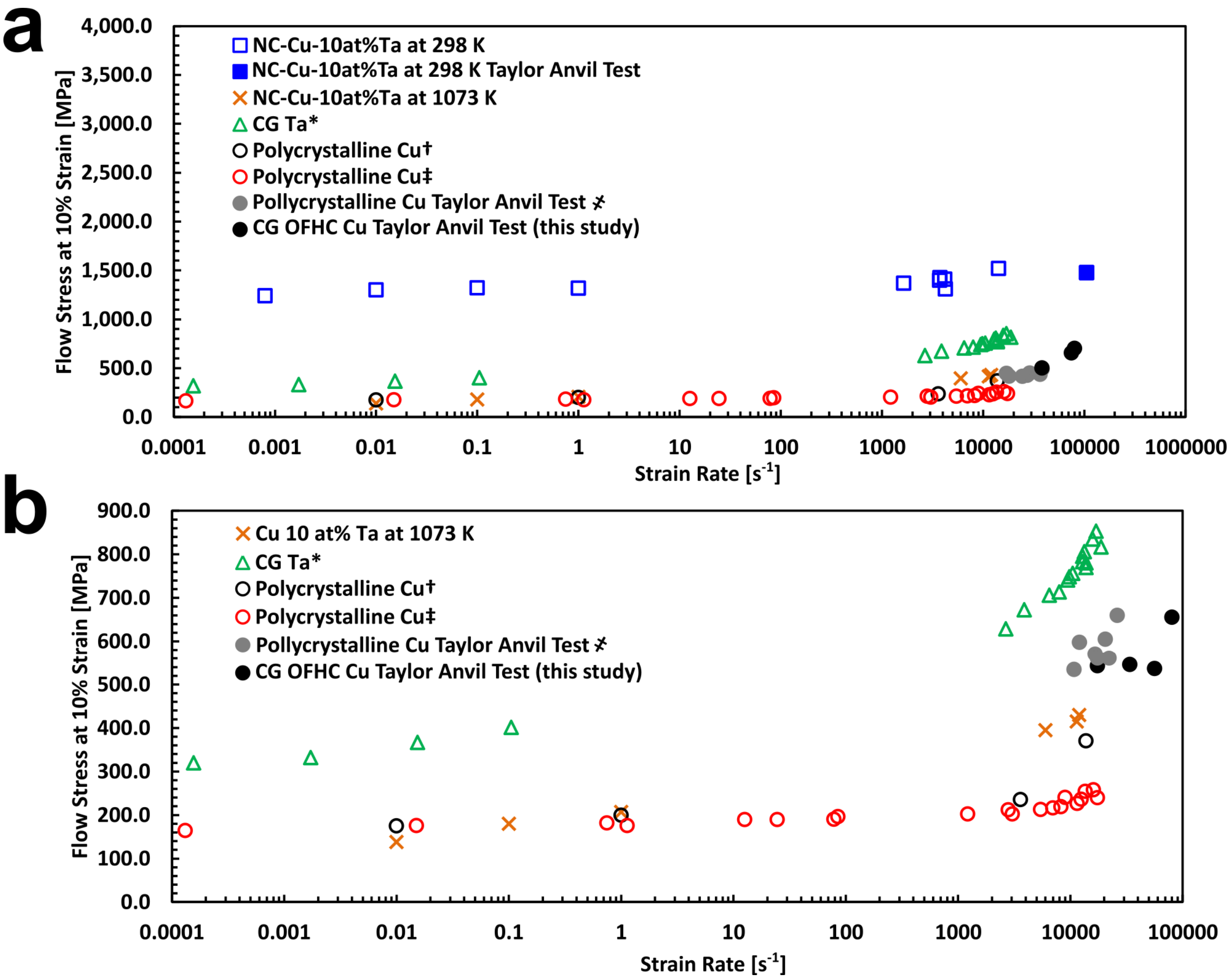

Supplementary Figure 8. The high flow stress of NC-Cu-10at.%Ta compared to that of various pure Cu and Ta materials. The plots of non-normalized flow stress both (a) with and (b) without NC-Cu-10at.%Ta indicate the immense increase in strength resulting from alloying and grain reduction even up to strain rates on the order of 10^5 s^{-1}. Data on polycrystalline and coarse grained (CG) materials are taken from Rittel et al. [9] (*), Jordan et al. [7](†), Follansbee and Kocks [8](‡), and House et al. [29] (≁). In this study, oxygen free high conductivity (OFHC) Cu was used as pure Cu for comparison with NC-Cu-10at.%Ta.

Similarly, Supplementary Figure 9, shown here to provide a comparison of the flow stress with respect to temperature at multiple strain rates, is similar to Figure 1B from the main document but using non-normalized flow stresses. Furthermore, as observed in the flow stress response of polycrystalline OFHC Cu, polycrystalline Ta, and NC-Cu as a function of strain rate, the flow stress above ~4×10^3 s^{-1} changes dramatically with a small increment in strain rate. To show that this upturn is a material phenomenon and not a result of geometrical effects, the method outlined by [7,30] for determining critical strain rates where inertia plays a role was followed.

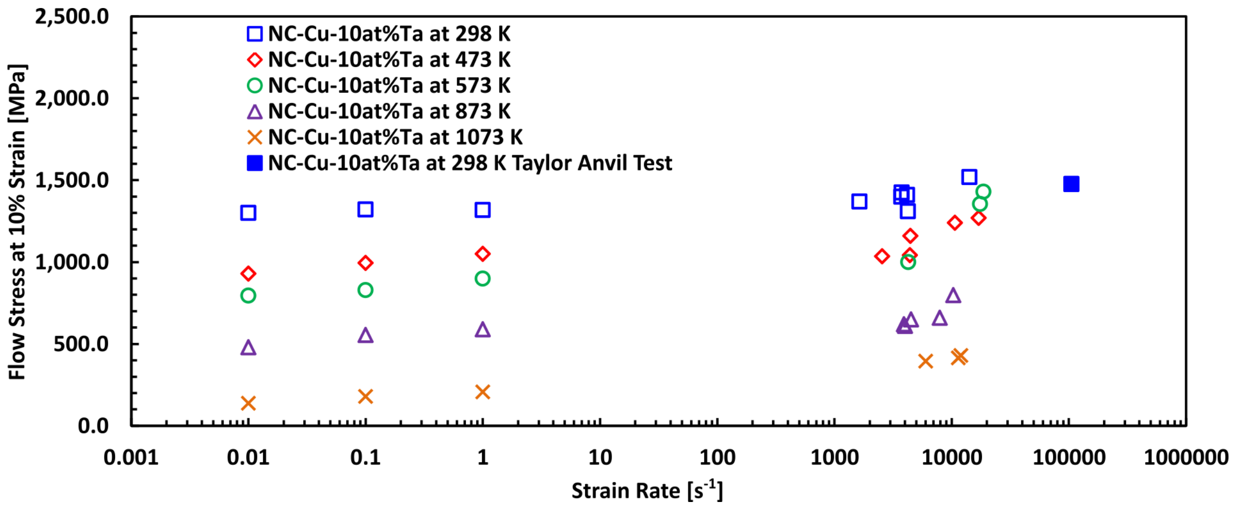

Supplementary Figure 9. The temperature response of the strain rate sensitivity of the flow stress in NC-Cu-10at.%Ta. Increasing temperature results in decreasing flow stress. In the case of samples tested at 573 K, flow stress begins to increase above that of 473 K beyond strain rates on the order of 10^4 s^{-1} indicating that drag effects become dominant sooner for the high temperature data than for the lower temperature data.

Supplementary Figure 10 shows that while the higher strain rate (> 1.2 x 10^4 s^{-1}) data from Follansbee and Kocks [8] could be influenced by inertia (see also [30]), the results obtained here for NC-Cu-10at.%Ta fall well below the critical strain rate for inertia. Further, Jordan et al.[7], who accounted for inertia effects, observe the upturn in flow stress in pure Cu at around 10^3 s^{-1}. In short, Follansbee and Kocks [8] observe the flow stress upturn at a higher strain rate than Jordan et al. [7] owing to detrimental inertia effects in the data of Follansbee and Kocks. However from the results of Jordan et al. with no inertia effects, the lack of flow stress upturn in NC-Cu-10at.%Ta can be determined a real material effect not influenced by specimen geometry.

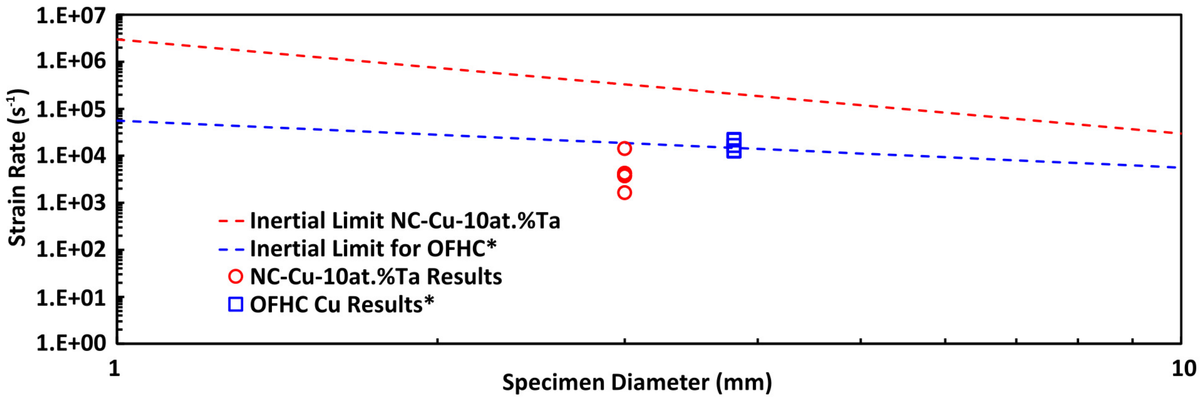

Supplementary Figure 10. Specimen size and strain rates compared to inertial limits for OFHC Cu and NC-Cu-10at.%Ta. While the data from Follansbee and Kocks [8](*) may be influenced by inertia at strain rates above 1.2 x 10^4 s^{-1}, the NC-Cu-10at.%Ta data all fall well below the strain rates where inertia plays a major role indicating that the flow stress is not significantly influenced by inertia effects.

Supplementary References

1. Segal, V. M. Materials processing by simple shear. *Mater. Sci. Eng. A* **197,** 157–164 (1995).
2. Furukawa, M., Horita, Z., Nemoto, M. & Langdon, T. G. Review: Processing of metals by equal-channel angular pressing. *J. Mater. Sci.* **36,** 2835–2843 (2001).
3. Zhu, Y. T. & Lowe, T. C. Observations and issues on mechanisms of grain refinement during ECAP process. *Mater. Sci. Eng. A* **291,** 46–53 (2000).
4. Kale, C. *et al.* On the roles of stress-triaxiality and strain-rate on the deformation behavior of AZ31 magnesium alloys. *Mater. Res. Lett.* **6,** 152–158 (2018).
5. Bacon, C., Carlsson, J. & Lataillade, J. L. Evaluation of force and particle velocity at the heated end of a rod subjected to impact loading. *J. Phys. IV* **01,** C3-395-C3-402 (1991).
6. Johnson, G., R. & Cook, W. H. A constitutive model and data for metals subjected to large strains, high strain rates and high temperatures. *Proc. 7th Int. Symp. Ballist.* **21,** 541–547 (1983).
7. Jordan, J. L., Siviour, C. R., Sunny, G., Bramlette, C. & Spowart, J. E. Strain rate-dependant mechanical properties of OFHC copper. *J. Mater. Sci.* **48,** 7134–7141 (2013).
8. Follansbee, P. S. & Kocks, U. F. A constitutive description of the deformation of copper based on the use of the mechanical threshold stress as an internal state variable. *Acta Metall.* **36,** 81–93 (1988).
9. Rittel, D., Bhattacharyya, A., Poon, B., Zhao, J. & Ravichandran, G. Thermomechanical characterization of pure polycrystalline tantalum. *Mater. Sci. Eng. A* **447,** 65–70 (2007).
10. Jones, S. E., Gillis, P. P. & Foster, J. C. On the equation of motion of the undeformed section of a Taylor impact specimen. *J. Appl. Phys.* **61,** 499–502 (1987).

11. Darling, K. A. *et al.* Extreme creep resistance in a microstructurally stable nanocrystalline alloy. *Nature* **537,** 378–381 (2016).

12. Rojhirunsakool, T. *et al.* Structure and thermal decomposition of a nanocrystalline mechanically alloyed supersaturated Cu–Ta solid solution. *MRS Commun.* **5,** 333–339 (2015).

13. Bhatia, M., Rajagopalan, M., Darling, K., Tschopp, M. & Solanki, K. The role of Ta on twinnability in nanocrystalline Cu–Ta alloys. *Mater. Res. Lett.* **5,** 48–54 (2017).

14. Chokshi, A., Rosen, A., Karch, J. & Gleiter, H. On the validity of the hall-petch relationship in nanocrystalline materials. *Scr. Metall.* **23,** 1679–1683 (1989).

15. Fougere, G., Weertman, J., Siegel, R. & Kim, S. Grain-size dependent hardening and softening of nanocrystalline Cu and Pd. *Scr. Metall. Mater.* **26,** 1879–1883 (1992).

16. Iyer, R. S., Frey, C. A., Sastry, S., Waller, B. & Buhro, W. Plastic deformation of nanocrystalline Cu and Cu–0.2 wt.% B. *Mater. Sci. Eng. A* **264,** 210–214 (1999).

17. Jiang, H., Zhu, Y. T., Butt, D. P., Alexandrov, I. V. & Lowe, T. C. Microstructural evolution, microhardness and thermal stability of HPT-processed Cu. *Mater. Sci. Eng. A* **290,** 128–138 (2000).

18. Sanders, P. G., Eastman, J. & Weertman, J. Elastic and tensile behavior of nanocrystalline copper and palladium. *Acta Mater.* **45,** 4019–4025 (1997).

19. Youssef, K. M., Scattergood, R. O., Linga Murty, K. & Koch, C. C. Ultratough nanocrystalline copper with a narrow grain size distribution. *Appl. Phys. Lett.* **85,** 929–931 (2004).

20. Agnew, S., Elliott, B., Youngdahl, C., Hemker, K. & Weertman, J. Microstructure and mechanical behavior of nanocrystalline metals. *Mater. Sci. Eng. A* **285,** 391–396 (2000).

21. Gray, G., Lowe, T., Cady, C., Valiev, R. & Aleksandrov, I. Influence of strain rate & temperature on the mechanical response of ultrafine-grained Cu, Ni, and Al-4Cu-0.5 Zr. *Nanostructured Mater.* **9,** 477–480 (1997).

22. Valiev, R. *et al.* Deformation behaviour of ultra-fine-grained copper. *Acta Metall. Mater.* **42,** 2467–2475 (1994).

23. Haouaoui, M., Karaman, I., Harwig, K. & Maier, H. Microstructure evolution and mechanical behavior of bulk copper obtained by consolidation of micro-and nanopowders using equal-channel angular extrusion. *Metall. Mater. Trans. A* **35,** 2935–2949 (2004).

24. Ebrahimi, F., Zhai, Q. & Kong, D. Deformation and fracture of electrodeposited copper. *Scr. Mater.* **39,** 315–321 (1998).

25. Lu, L., Shen, Y., Chen, X., Qian, L. & Lu, K. Ultrahigh strength and high electrical conductivity in copper. *Science* **304,** 422–426 (2004).

26. Ma, E. Instabilities and ductility of nanocrystalline and ultrafine-grained metals. *Scr. Mater.* **49,** 663–668 (2003).

27. Chawla, K. K. & Meyers, M. *Mechanical behavior of materials*. (Prentice Hall, 1999).

28. Hayashi, K. & Etoh, H. Pressure sintering of iron, cobalt, nickel and copper ultrafine powders and the crystal grain size and hardness of the compacts. *Mater. Trans. JIM* **30,** 925–931 (1989).

29. House, J. W., Lewis, J. C., Gillis, P. P. & Wilson, L. L. Estimation of flow stress under high rate plastic deformation. *Int. J. Impact Eng.* **16,** 189–200 (1995).

30. Gorham, D. A. The effect of specimen dimensions on high strain rate compression measurements of copper. *J. Phys. Appl. Phys.* **24,** 1489 (1991).
